# Recurrent bacterial meningitis caused by incomplete Type I inner ear malformation: A case report

**DOI:** 10.1002/ibra.12093

**Published:** 2023-02-13

**Authors:** Zhong Luo, Piao Cao, Chun‐Lin Zhang, Zu‐Cai Xu, Ping Xu, Tao Liang

**Affiliations:** ^1^ Department of Neurology Affiliated Hospital of Zunyi Medical University Zunyi Guizhou China; ^2^ Department of Otorhinolaryngology Affiliated Hospital of Zunyi Medical University Zunyi Guizhou China

**Keywords:** bacterial meningitis, case report, incomplete Type I inner ear malformation

## Abstract

The incidence of incomplete partition Type I inner ear malformation is very low; therefore, bacterial meningitis caused by this malformation is also rare. Here, we report a case of such a patient. This case is a young female patient, who is 7 years old, began to have recurrent headaches, and after 5 years, also began to have chest and back pain. The doctor diagnosed meningitis, and the anti‐infection treatment was effective. She was followed up annually and continued to have outbreaks repeatedly for 17 years, but the cause of repeated infection was not found. After a detailed diagnosis and treatment in our hospital, the patient was finally diagnosed with incomplete partition Type I inner ear malformation, resulting in repeated bacterial meningitis. The patient recovered well after surgical treatment, and the symptoms did not recur after 1‐year follow‐up.

## Graphical abstract

This case is a young female patient. When she was 7 years old, she began to have recurrent headaches, then, after 5 years, she also began to have chest and back pain. The doctor diagnosed meningitis, and the anti‐infection treatment was effective. The headaches occurred once a year and continued to break out repeatedly for 17 years, but the cause of repeated infection has not been found. After detailed diagnosis and treatment in our hospital, it was finally diagnosed as incomplete separation Type I inner ear malformation, resulting in repeated bacterial meningitis. The patient recovered well after surgical treatment, and the symptoms did not recur after 1 year of follow‐up.

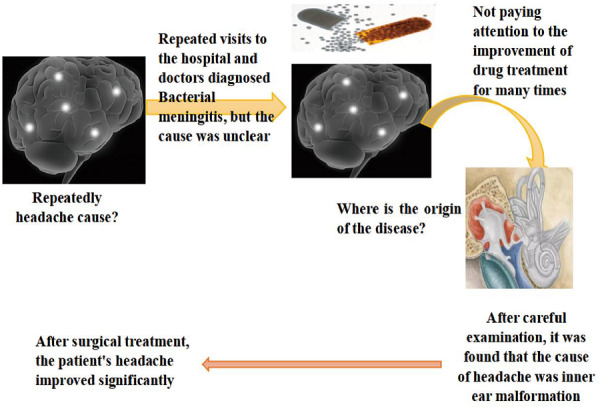

## 1 INTRODUCTION

Purulent meningitis is a central nervous system infection caused by different bacteria, and both the brain and spinal cord can be involved. In recent years, the incidence of purulent meningitis in children has been higher than in adults.^1^, ^2^, ^3^, ^4^ Therefore, it is of great importance for suppurative meningitis in adults, especially recurrent meningitis, to find and treat the cause of recurrent infection. Cerebrospinal fluid (CSF) leakage is a common indication of skull base lesions. However, not all patients with recurrent meningitis have this symptom. Here, we report a case of recurrent meningitis caused by bacterial infection. The patient was finally diagnosed with recurrent intracranial infection caused by CSF otorrhea and incomplete partition Type I inner ear malformation. The purpose of our study is to bring attention to and enhance the general understanding of this disease.

## 2 CASE PRESENTATION

**Figure 1:**
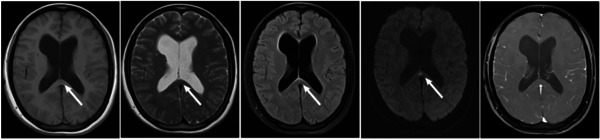
On September 18, brain magnetic resonance imaging image revealed that there were flake long T2 signals in the right basal ganglia, FLAIR showed low signals, small flake long T2 signals in the splenium of corpus callosum, DWI showed high signals (white arrow), and there was no enhancement on enhanced examination.

**Figure 2:**
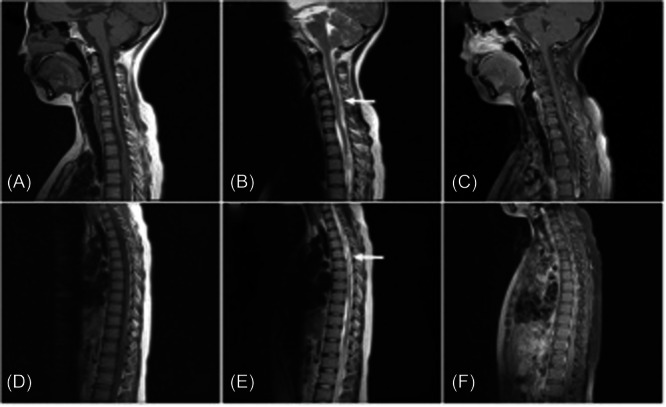
On September 18, an magnetic resonance imaging of the spinal cord displayed that multiple patchy mixed long T2 signal shadows were seen under the extramedullary epidural in the spinal canal from the lower edge of cervical vertebra 5 to thoracic vertebra 5, and the spinal cord was slightly deformed and displaced under compression (A–C). From the level of cervical vertebra 7 to thoracic vertebra 12, multiple patchy mixed long T2 signals were seen under intradural extramedullary, and patchy slightly long T2 signals were seen in the spinal cord of thoracic vertebra 3–5 (D–F). [Correction added on 20 May 2024, after first online publication: Figure 2 was revised in this version at the request of the authors.]

**Figure 3:**
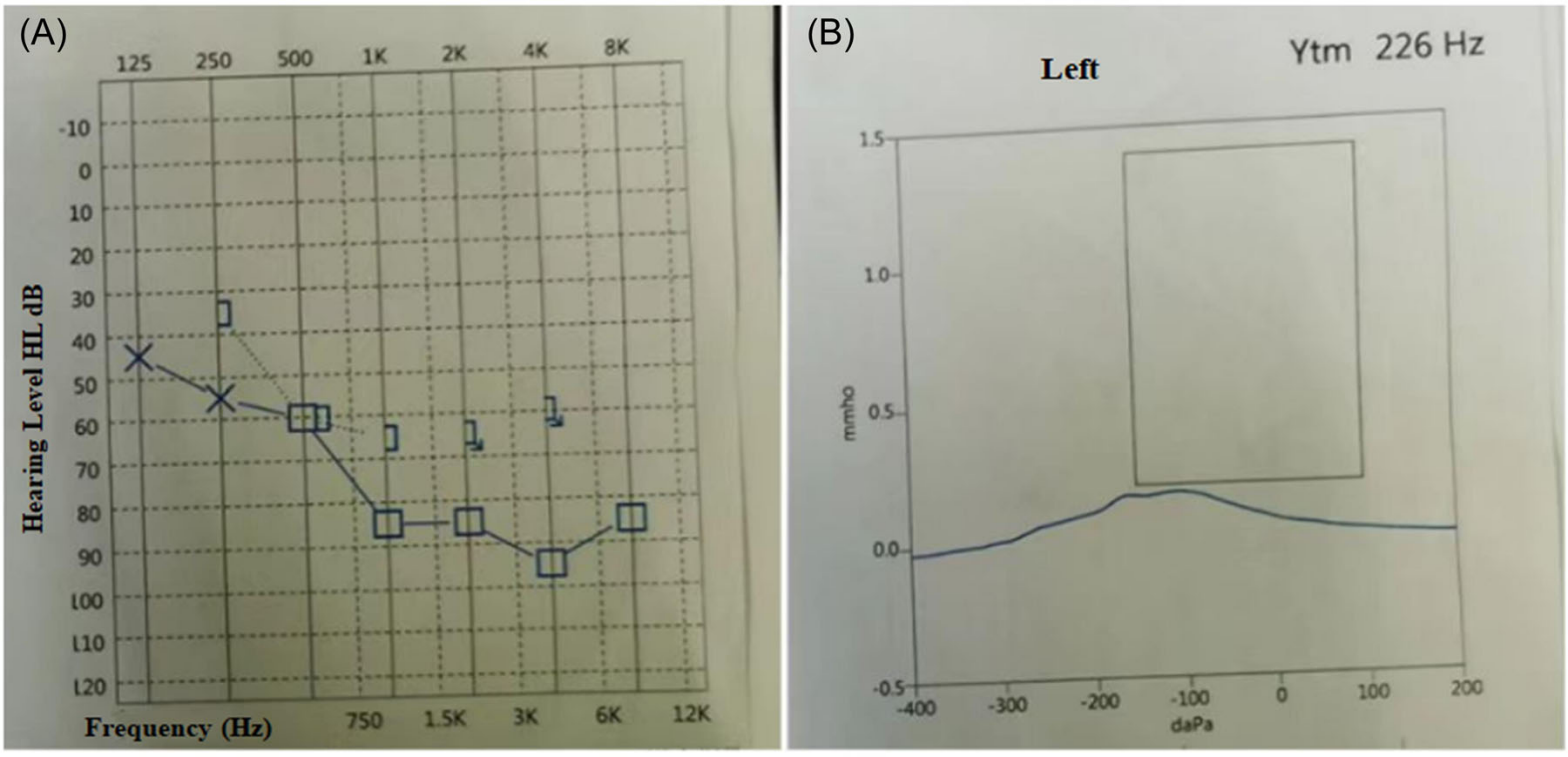
The patient's electroaudiometry (A) indicated decreased hearing in her left ear, and acoustic impedance (B) prompted Type B diagram. [Color figure can be viewed at wileyonlinelibrary.com]

**Figure 4:**
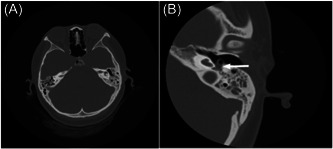
High‐resolution chest tomography of temporal bone found that the density of the left mastoid air chamber increased, a few heightened density shadow was found in the tympanic chamber, and the left internal auditory canal was narrower than the right.

**Figure 5:**
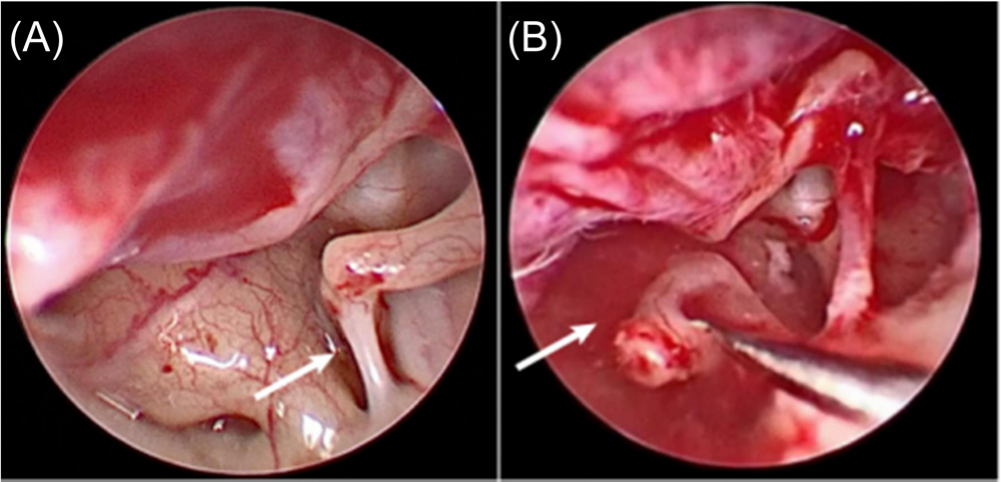
During the operation, cerebrospinal fluid exudation was seen around the stapes (A), and cerebrospinal fluid blowout occurred after removing the stapes (B). [Color figure can be viewed at wileyonlinelibrary.com]

A 24‐year‐old female was hospitalized because of recurrent headache with chest and back pain. At the age of 7, the patient had recurrent headache with no obvious cause, and the main manifestations were persistent distending pain in the forehead and occipital region, accompanied by fever. Once at a visit to a local hospital, the patient complained of meningitis after a relevant examination, and her headache was completely relieved after infusion treatment (the exact details of the specific drug were not given) for approximately 1 week. However, the headache continued to occur approximately once a year and was alleviated after infusion each time. At the age of 12, the patient had a headache with chest and back severe tingling and burning pain, which could radiate to both upper limbs. The above symptoms occurred intermittently and repeatedly, lasting from a few seconds to a few minutes each time. Fever could attack dozens of times a day. Her left hearing gradually decreased when she was 13 years old, but she did not attach great importance to this or seek treatment. More than a year later, the above symptoms worsened again, accompanied by a coma (details unknown), and she immediately attended the local hospital, where tuberculous meningitis was considered. Therefore, the hospital gave the patient regular antituberculosis treatment (isoniazid, rifampicin, pyrazinamide, and ethambutol). After maintenance treatment for more than 1 year, the above symptoms were in remission, and she did not come back to the hospital for follow‐up. The patient stopped taking the drugs on her own approximately 7 months before admission. Three days before admission, the above symptoms recurred after the patient had a cold; subsequently, she measured her temperature up to 37.8°C. Seeking further diagnosis and treatment, she was treated in our hospital and admitted to our department. We also considered recurrent meningitis after asking about the patient's relevant medical history and physical examination, but the patient told us that she had no CSF rhinorrhea or otorrhea. Neurological examinations showed that the neck strength was 2 fingers, and Kernig and Brudzinski proved negative. Left hearing loss was found after a rough measurement of the patient's hearing. All other physical examinations were unremarkable. The supplementary examinations were as follows. Computed Tomography (CT) of the brain revealed right basal ganglia encephalomalacia and supratentorial hydrocephalus. Routine blood examination showed that the total number of leukocytes was 13.71 ×  10^9^/L, and the absolute value of neutrophils was 11.65 × 10^9^/L. C‐reactive protein was 57.30 mg/L. Treatment teams considered that the patient had a previous history of meningitis that was alleviated after short‐term infusion (probably antibiotics) and antituberculosis drug treatment; therefore, it was considered that there was a high possibility of recurrent intracranial infection or tuberculosis recurrence and drug resistance. Therefore, on September 16, CSF was collected via lumbar puncture, and the test results are shown in Table 1. Suppurative meningitis was diagnosed, and ceftriaxone sodium (4 g per day) was used for anti‐infection therapy. After that, magnetic resonance imaging (MRI) of the brain showed old lacunar infarction and malacia in the bilateral basal ganglia, an abnormal signal of the splenium of the corpus callosum and supratentorial hydrocephalus (Figure 1). MRI of the spinal cord suggested that from the lower edge of the fifth cervical vertebra to the level of the fifth thoracic vertebra, there were multiple intraspinal extramedullary subdural lesions, slight edema of the spinal cord at thoracic vertebrae 3–5 and compression of the spinal cord. There were multiple extramedullary subdural lesions at the level of cervical vertebra 7 to thoracic vertebra 12, and the spinal cord signal of thoracic vertebrae 3–5 was abnormal. Infectious lesions were taken into account (Figure 2). After 3 days, high‐throughput gene detection of pathogenic microorganisms (PMseq‐DNA) showed group G + Streptococcus. Therefore, we added vancomycin (1.5 g/day) combined with anti‐infection treatment, and betamethasone sodium phosphate injection (10.52 mg/day) was used to relieve the inflammatory reaction. After 3 days of combination therapy, a CSF reexamination revealed obvious decreases in leukocytes and protein (Table 1). The patient reported headache remission, and there was no recurrent chest or back pain, suggesting that the treatment was effective. Therefore, the diagnosis of suppurative meningitis was determined. However, the patient had encephalitis repeatedly over the past 10 years, and there should be a cause of bacterial infection. We repeatedly asked whether there were symptoms or manifestations of CSF otorrhea or rhinorrhea, but the patient denied this case history. The treatment teams noticed that the left ear hearing had gradually decreased after the patient was 13 years old. After consulting the otorhinolaryngology department, they found that the patient had no deformity of the auricle, a complete tympanic membrane, and no obvious hydrops. Pure tone audiometry and acoustic immittance audiometry were performed, suggesting neurological deafness (Figure 3). After improving the examination of the temporal bone using high‐resolution chest tomography (HRCT), it was found that the density of the left mastoid air chamber was increased, a few heightened density shadows were found in the tympanic chamber, and the left internal auditory canal was narrower than the right (Figure 4). Hence, the otorhinolaryngology department was consulted again; the possibility of congenital inner ear malformation was considered, and they recommended surgery within a specified time limit. The patient's clinical symptoms actively achieved complete remission with antibiotic therapy. CSF examination showed that the number of leukocytes and proteins was markedly lower than before and close to normal. Reexamination of the brain MRI showed that the lesions in the splenium of the corpus callosum had disappeared, and the MRI of the spinal cord found that there was no significant change compared with before. After the patient returned from the hospital, she completed the preoperative examination in the otorhinolaryngology department of our hospital on November 27; then, surgery comprising left stapes resection under an otoendoscope, fenestration of the inner ear, repair of CSF otorrhea, and removal of the temporal muscle was performed. During the operation, we found that the posterior part of the stapes footplate sustained CSF leakage (Figure 5). After surgery, the CSF no longer exuded. The patient was diagnosed with an incomplete partition Type I inner ear malformation. She was followed up for 1 year without recurrent headaches or paroxysmal chest or back pain.

**Table 1:** Cerebrospinal fluid examination results during hospitalization.

Item	Sept 16	Sept 19	Sept 22	Sept 29	Oct 9
Pressure	70 mm H_2_O	115 mm H_2_O	80 mm H_2_O	100 mm H_2_O	80 mm H_2_O
Appearance	Yellow turbidity	Colorless transparent	Colorless transparent	Colorless transparent	Colorless transparent
Total cell count	7776 × 10^6^/L	117 × 10^6^/L	60 × 10^6^/L	6 × 10^6^/L	108 × 10^6^/L
Leukocyte count	4134 × 10^6^/L	30 × 10^6^/L	21 × 10^6^/L	1 × 10^6^/L	3 × 10^6^/L
Chlorine	119.6 mmol/L	125.9 mmol/L	128.1 mmol/L	123.7 mmol/L	123.7 mmol/L
Glucose	2.96 mmol/L	2.57 mmol/L	4.43 mmol/L	4.69 mmol/L	4.46 mmol/L
LDH	49 U/L	34 U/L	26 U/L	23 U/L	26 U/L
CSF protein quantity	4302 mg/L	1162 mg/L	767 mg/L	681 mg/L	511 mg/L
Adenosine dehydrogenase	0.99 U/L	3.38 U/L	1.19 U/L	0.26 U/L	0.09 U/L

Abbreviations: CSF, cerebrospinal fluid; LDH, lactate dehydrogenase.

## 3 DISCUSSION

**Figure 6:**
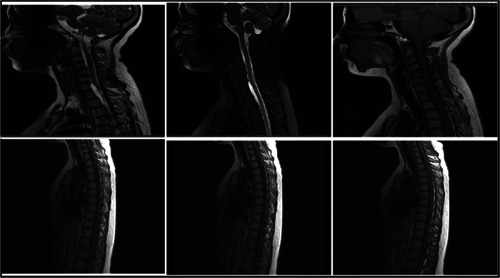
The C (4–7) showed mild lumbar disc herniation, and fusiform water signal intensity can be seen in front of the horizontal spinal cord of C7–T2. Multiple patchy mixed long T2 signals as the main abnormal signals were found at the level of extramedullary subdural of C7–T12. At the T3–5 level, a slightly longer T2 signal was seen in the spinal cord.

Internal ear malformation is prone to cause recurrent meningitis.^5^, ^6^ In this case report, the patient had recurrent episodes of meningitis for 17 years, through continuous imaging examinations and with the help of otolaryngologists, it was finally found that the patient's diagnosis was IP‐I, and after surgical treatment, the patient recovered well. After 1 year of follow‐up, there was no recurrence of the patient's headache or paroxysmal chest or back pain. The results of the MRI reexamination revealed no intracranial lesions, but the spinal cord lesions did not completely disappear (Figure 6).

The recognized classification of inner ear malformation is based on the imaging findings of the temporal bone. Sennaroglu et al.^7^ divided inner ear deformities into complete labyrinthine aplasia (Michel deformity), cochlear aplasia, cochlear hypoplasia, incomplete partition (Mondini deformity), and common cavity. Subsequently, because of an increased frequency of CT scans of the ear, which are performed before cochlear implantations, Sennaroglu and Saatci made some amendments to the classification and added terms for two different incomplete partition deformities: IP‐I (cystic cochleovestibular malformity) and IP‐II (the classic Mondini deformity). Congenital malformation of the inner ear is rare in the clinic and is one of the common causes of recurrent bacterial meningitis. Incomplete partition is a subtype of bone labyrinth deformity among inner ear deformities,^8^ accounting for approximately 20% of sensorineural hearing loss; moreover, IP‐I inner ear malformation is rare. According to the literature, IP‐I malformation accounts for approximately 4.5%–8.7% of inner ear malformations,^7^, ^9^, ^10^ and on CT images, the morphology of the cochlea and the semicircular canal is basically normal, and only the vestibular cavity is enlarged. Therefore, when the disease occurs, the lesion is hard to find. For such patients, in addition to accurate imaging evaluations, if there are repeated clinical manifestations of intracranial infection, great emphasis should be placed on the possibility of inner ear malformation. Park et al. found that the recognition time of inner ear malformations was 25.7 months,^11^ and radiographic analyses included HRCT and MRI.^12^, ^13^ The patient was finally diagnosed with IP‐I of the left inner ear malformation by HRCT of the temporal bone and surgical observations. Although IP‐I is not the most common inner ear malformation, it will be encountered in clinical practice, and owing to defects in the stapes floor, it most often causes meningitis; however, CSF otorrhea and rhinorrhea are not common, therefore making it difficult to diagnose. It was found that 21 of the 39 patients with IP‐I treated by CI at Hacettepe University did not have CSF leakage during the operation.^14^, ^15^

The main treatment for inner ear malformation is surgery. Ying Shi et al.^12^ conducted a retrospective study on 877 children with inner ear malformations after surgery. In their paper, multiple CT scans before and after surgery showed that 754 of the cases (86.0%) might achieve satisfactory effects. Moreover, if the hearing does not recover after surgery, cochlear implantation is feasible and is a relatively safe and effective treatment for Type I incomplete partition.^17^ Although the incidence of cochlear revision surgery is 4.23%, it has been shown that patients' hearing and language performance improves after revision surgery, indicating that this operation is effective in most cases.^18^ Therefore, for recurrent meningitis, finding the etiology and active treatment is the key to relieving patients' pain. Our hospital also reported a case of meningitis caused by IP‐II. Over time, we believe that additional types of meningitis caused by inner ear malformations will be reported and gradually recognized.

## AUTHOR CONTRIBUTIONS

Tao Liang and Piao Cao conceptualized and designed the study, drafted the initial manuscript, and reviewed and revised the manuscript. Zhong Luo and Zu‐Cai Xu were responsible for medical data collection and reviewed and revised the manuscript. Chun‐Lin Zhang performed surgical treatment and revised the manuscript. Ping Xu developed a treatment plan, designed the data collection instruments, coordinated and supervised data collection, and critically reviewed the manuscript. All authors contributed to the interpretation of the findings and critical revision of the manuscript and approved the final manuscript.

## CONFLICT OF INTEREST STATEMENT

The authors declare no conflict of interest.

## ETHICS STATEMENT

This study was approved by the Ethics Committee of ZunYi Medical University (Approval No. KLL‐2022‐774). The informed consent was obtained from the patient.

## Data Availability

Data are available on request from the authors.
